# Myocardial perfusion imaging performed by dedicated cardiac cadmium-zinc-telluride camera in dextrocardia with situs inversus: A case report

**DOI:** 10.1007/s12350-021-02696-6

**Published:** 2021-06-24

**Authors:** Lena Forsberg, Mariana Soares, Adrian Gonon

**Affiliations:** 1 Department of Clinical Physiology, Karolinska University Hospital, 171 76 Stockholm, Sweden; 2 Department of Molecular Medicine and Surgery, Karolinska Institutet, Stockholm, Sweden; 3 Division of Clinical Physiology, Department of Laboratory Medicine, Karolinska Institutet, Stockholm, Sweden

## Introduction

Dextrocardia with situs inversus is a rare congenital condition with a prevalence of 1:10,000. There are few studies in the literature that have described myocardial perfusion imaging (MPI) in this patient population and the publications are mainly case reports.^1^

The current case is presented in order to show that cadmium-zinc-telluride (CZT) camera especially dedicated for the heart can be used to perform MPI in patients with dextrocardia.

## Case Report

**Figure 1:**
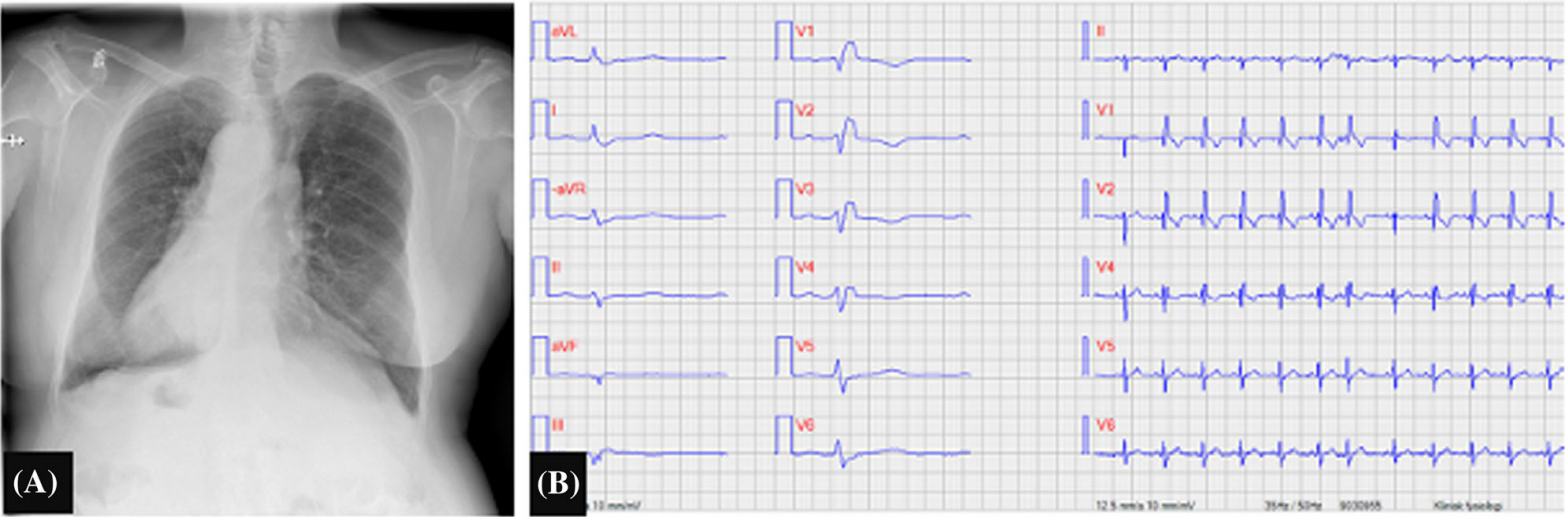
**A** Chest X-ray showing dextrocardia. **B** Electrocardiogram (ECG) with reversed leads showing right bundle branch block and left anterior hemiblock

A 77-year old male with known dextrocardia in situs in versus and hypertension was referred to our clinic to investigate whether his experienced symptoms were related to myocardial ischemia (Figure 1). The patient was examined according to a two-day stress-rest protocol with regadenoson in combination with bicycle ergometry. Technetium (99mTc) tetrofosmin ^99m^Tc tetrofosmin (639 MBq) was injected 30 seconds after regadenoson was administrated.^2^

**Figure 2:**
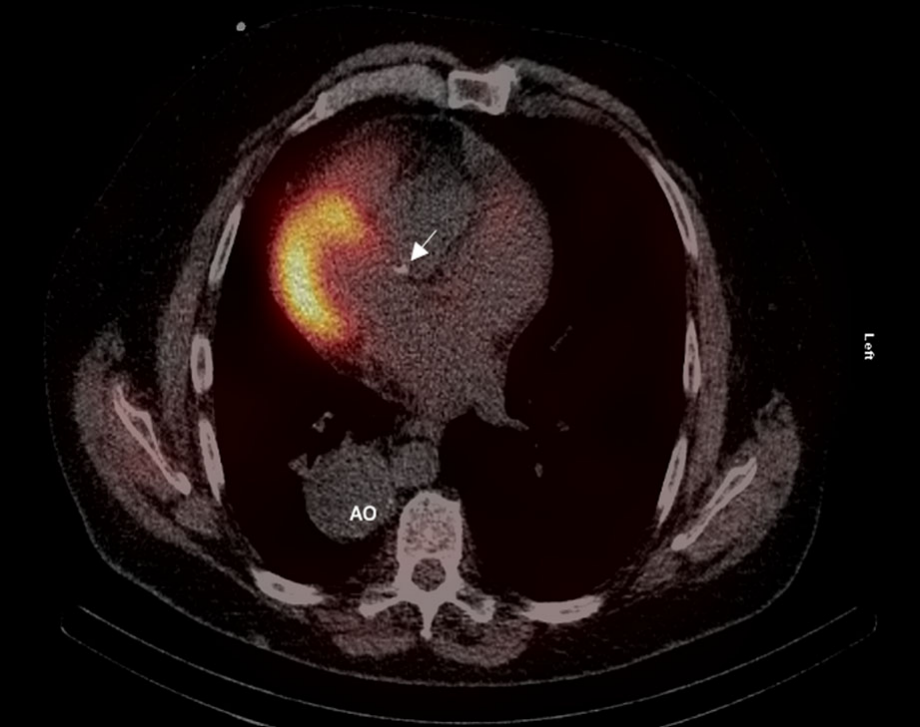
SPECT/CT of the patient demonstrating dextrocardia with situs inversus. A calcified plaque in the proximal left anterior descending artery is indicated by an arrow. The thoracic aorta is slightly dilated

**Figure 3:**
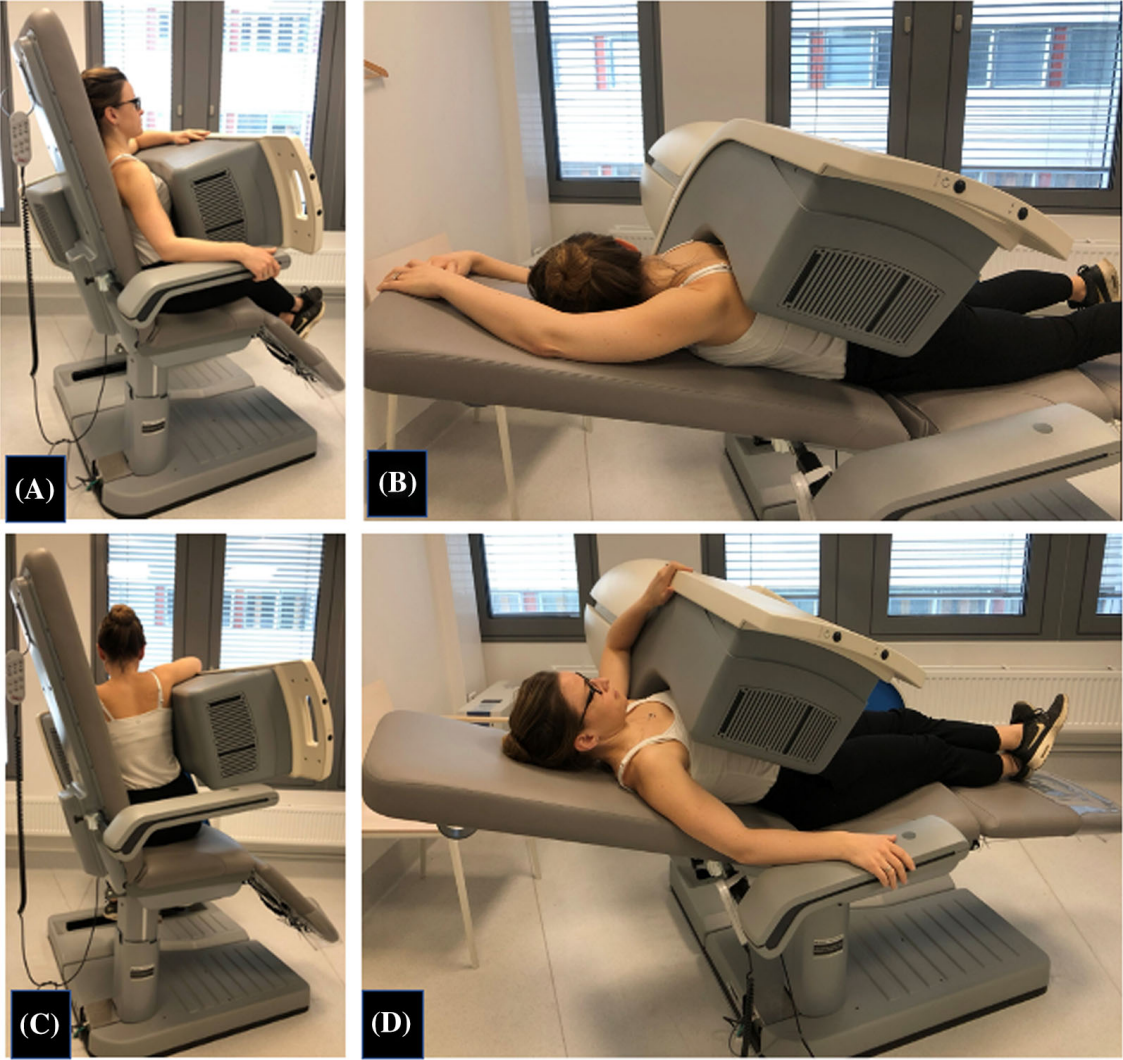
In the D-SPECT patients with normal position of the heart (levocardia) are usually examined in both supine and upright position to enable assessment of attenuation: **A** upright position **B** Supine position. In the patient with dextrocardia the position in the chair had to be adjusted so the heart could correctly be centered. **C** Upright positioning at approximately 70° chair angle the heart could be centered within the positioning ellipse. The patient can still sit comfortably by supporting the arms on top of the detector and leaning towards the backrest. **D** By lowering the backrest at approximately 20° chair angle the patient can be in a prone position, and with a tight positioning of the detector arm around the patients back the left ventricle can be centered within the positioning ellipse

**Figure 4:**
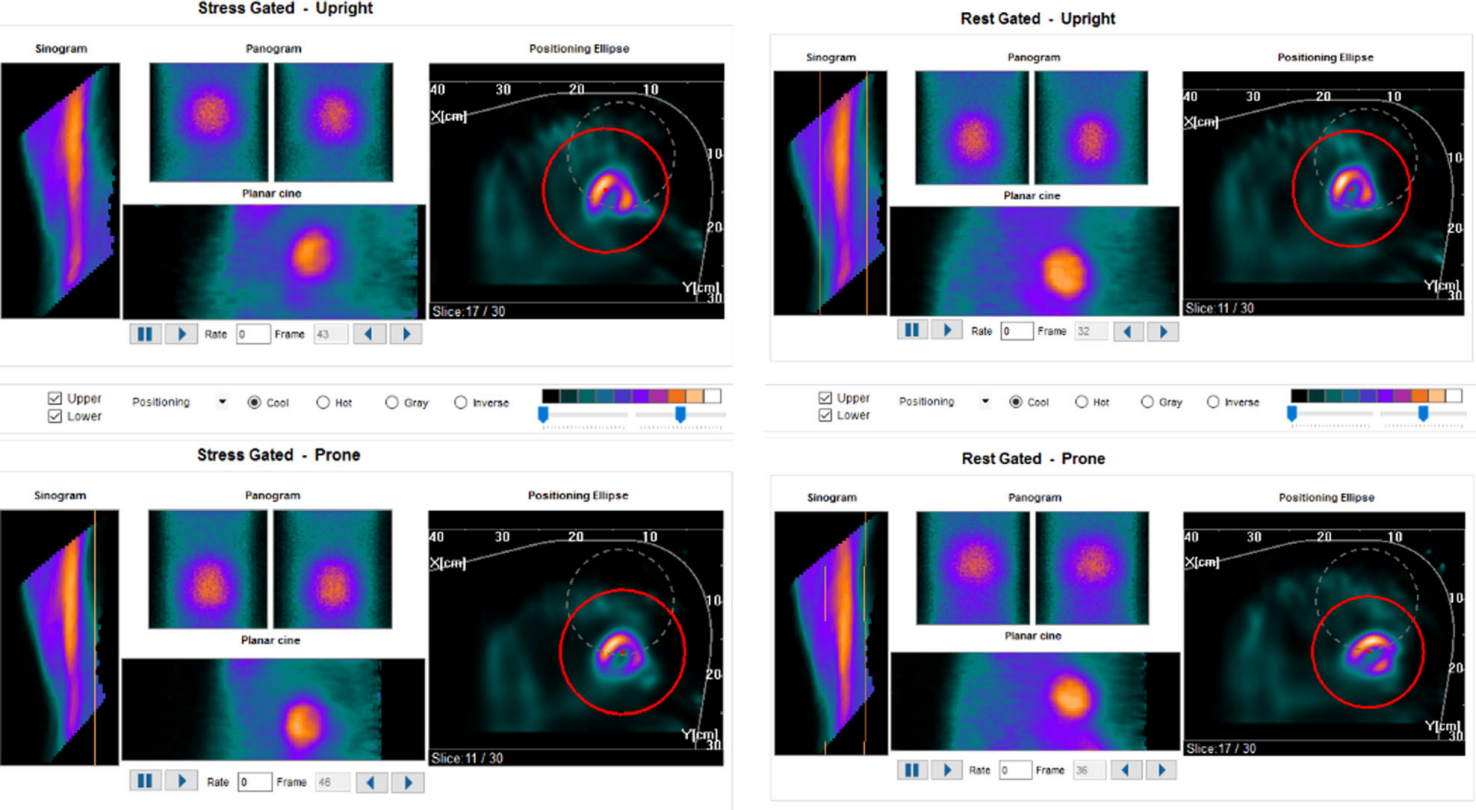
Data quality control report from the D-SPECT with the upright position at the top row and the prone position at the bottom row, stress and rest. The sonogram and panogram shows no movement during the scans. In the image named positioning ellipse the distance between the detector arm and the left ventricle is presented and the left ventricle has unchanged relation to the detector arm at both stress and rest

**Figure 5:**
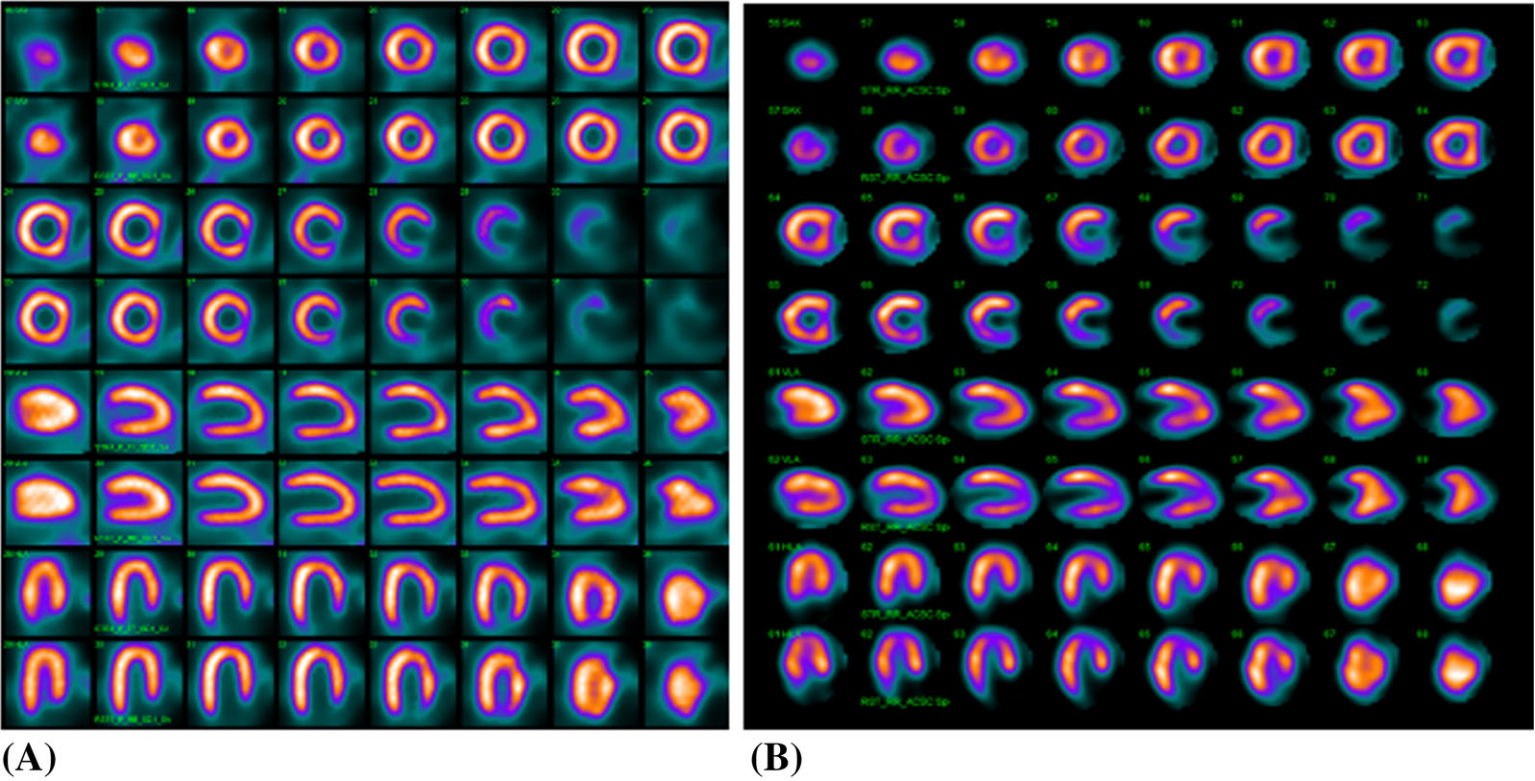
**A** ^99m^Tc tetrofosmin images in prone position at stress and rest reconstructed with iterative algorithm processing showing the right ventricle where the left ventricle is usually seen and therefore the myocardial septal and lateral walls have another location (mirror images). In the vertical long axis (VLA) view the anterior and inferior wall have not changed place. **B** ^99m^Tc tetrofosmin images in SPECT with CT attenuation correction

MPI were first acquired by a single photon emission tomography/ computed tomography (SPECT/CT; Symbia T16, Siemens Healthineeers, Erlangen, Germany) equipped with low energy high resolution collimator and MPI were reconstructed by a specific software for dextrocardia (HERMES Medical Solutions, Stockholm, Sweden) (Figure 2). Another scan was consecutively performed within one hour with a cadmium-zinc-telluride SPECT with cardiac dedicated detector (D-SPECT, Spectrum Dynamics). In the cardiac dedicated camera, the patient was positioned in atypical position to have the heart in focus of the nine detector columns (Figures 3, 4) Rest images were obtained a few days later with the same acquisition protocol. MPI at stress and rest of both cameras presented a high quality of acquisition. Both scanners presented comparable and typical myocardial perfusion pattern without any signs of decreased uptake (Figure 5).

## Conclusion

Hereby, we demonstrated that D-SPECT can reliable perform MPI in dextrocardia with situs inversus by a specific positioning of the body in the gantry.
